# Diffusion‐Free Scaling in Rotating Spherical Rayleigh‐Bénard Convection

**DOI:** 10.1029/2021GL095017

**Published:** 2021-10-21

**Authors:** Guiquan Wang, Luca Santelli, Detlef Lohse, Roberto Verzicco, Richard J. A. M. Stevens

**Affiliations:** ^1^ Department of Science and Technology Physics of Fluids Group and Twente Max Planck Center, Mesa+ Institute J. M. Burgers Center for Fluid Dynamics University of Twente Enschede The Netherlands; ^2^ Gran Sasso Science Institute L'Aquila Italy; ^3^ Max Planck Institute for Dynamics and Self‐Organization Göttingen Germany; ^4^ Dipartimento di Ingegneria Industriale University of Rome’ Tor Vergata’ Rome Italy

**Keywords:** Thermal convection, spherical shell, rapidly rotating, diffusion‐free scaling

## Abstract

Direct numerical simulations are employed to reveal three distinctly different flow regions in rotating spherical Rayleigh‐Bénard convection. In the high‐latitude region I vertical (parallel to the axis of rotation) convective columns are generated between the hot inner and the cold outer sphere. The mid‐latitude region II is dominated by vertically aligned convective columns formed between the Northern and Southern hemispheres of the outer sphere. The diffusion‐free scaling, which indicates bulk‐dominated convection, originates from this mid‐latitude region. In the equator region III, the vortices are affected by the outer spherical boundary and are much shorter than in region II.

## Key Points

- We show that in rotating spherical Rayleigh‐Bénard convection, three regions with distinctly different flow dynamics are formed
- The mid‐latitude region is characterized by convective columns that extend from the Northern to the Southern hemisphere of the outer sphere
- The diffusion‐free scaling indicates that the flow dynamics and heat transport originating in the mid‐latitude region are bulk‐dominated

## 1 Introduction

**Figure 1:**
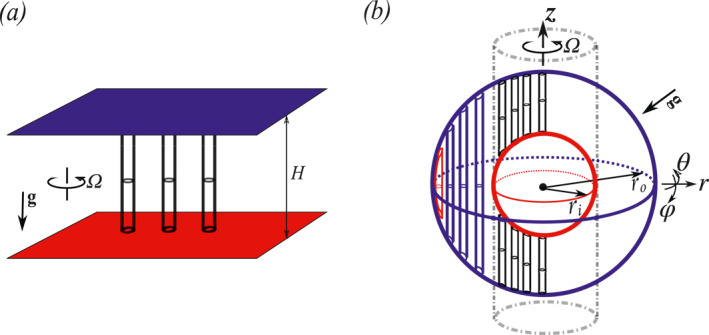
Schematics showing the alignment of the axial convective columns in (a) canonical framework heated from bottom and cooled from above and (b) spherical rotating RB convection heated from inner and cooled from outer, in which the gravity points toward the geometrical center. The longitudinal (azimuthal), co‐latitudinal (polar), and radial directions are represented by $\hat{\theta }$, $\hat{\varphi }$ and $\hat{r}$, respectively. The rotation axis aligns with the z‐direction. The angle between gravity and rotation axis is φ. The tangent cylinder is shown with dashed‐dotted gray line. Panel (b) is adapted from Aurnou et al. (2015) and Busse (1970, 1983).

Rapidly rotating convection is relevant for many geophysical and astrophysical flows, e.g., the solar interior (Schumacher & Sreenivasan, 2020), the liquid metal core of terrestrial planets (Aurnou et al., 2015; Jones, 2011; Olson, 2011; Zhang & Schubert, 2000), and Earth's oceans and atmosphere (Fultz et al., 1959; Marshall & Schott, 1999). In these instances of convection with strong thermal driving, the flow dynamics is nevertheless dominated by the strong background rotation (Aurnou et al., 2015; Kunnen, 2021; Sprague et al., 2006). The effect of rotation has been extensively studied in Rayleigh‐Bénard (RB) convection experiments (Cheng et al., 2020; Ecke & Niemela, 2014; King et al., 2009, 2012; Liu & Ecke, 1997; Rossby, 1969; Stellmach et al., 2014; Stevens et al., 2009; Wedi et al., 2021; Zhong et al., 2009) and simulations (Horn & Shishkina, 2015; King et al., 2012, 2013, 2009; Kunnen et al., 2016; Schmitz & Tilgner, 2009; Stellmach et al., 2014; Stevens et al., 2009). In the canonical RB system, the flow is confined between two parallel plates, and this system is studied in 3D periodic, rectangular, or cylindrical domains. In the remainder of this article, we refer to this as planar RB convection to distinguish it from the spherical RB system considered here (see Figure 1a). We refer the reader to the reviews (Aurnou et al., 2015; Kunnen, 2021; Plumley & Julien, 2019) for an extensive explanation of rotating RB convection. Even though there are great community efforts on rotating RB the diffusion‐free scaling regime, geostrophic dominated which will be defined explicitly below, predicted for strongly thermally driven rotation dominated flow has not been observed yet for rotating RB with no‐slip boundaries. This study will show that in a spherical RB convection, the geometry allows for the formation of a geostrophic dominated flow region that exhibits diffusion‐free scaling in the mid‐latitude region.

The control parameters of rotating RB flow are the Rayleigh (Ra), Ekman (Ek), and Prandtl (Pr) numbers, to be defined explicitly below. Derived from these, the convective Rossby number $Ro\equiv \sqrt{Ra/Pr}Ek/2$ characterizes the importance of the thermal forcing relative to rotation (Gilman, 1977). With increasing Rayleigh number Ra and for strong rotation Ro≪1, two regimes can be identified, namely: (a) the *weakly nonlinear regime* for Ra near the onset of convection, (b) the *quasi‐geostrophic regime* for $Ra/Ra_{c}\leq 3$ (Ecke & Niemela, 2014), where $Ra_{c}∼Ek^{-4/3}$ is the critical Rayleigh number for the onset of convection (Chandrasekhar, 1961). In a third regime (c), for Ro≫1 and high enough Ra, the flow approaches the non‐rotating RB convection case (Ahlers et al., 2009; Chilla & Schumacher, 2012; Grossmann & Lohse, 2000).

For the quasi‐geostrophic regime, when Ek→0, the Nusselt number Nu (i.e., the non‐dimensional heat transfer) is found to depend on the supercriticality $Nu∼Pr^{\gamma }{\left(Ra/Ra_{c}\right)}^{\alpha }$ (Cheng et al., 2015; Julien, Knobloch, et al., 2012; King et al., 2012; Stellmach et al., 2014). When the heat transport is independent of molecular diffusion in the asymptotic limit, this results in α=3/2 and γ=−1/2. This scaling $Nu∼Pr^{-1/2}{\left(Ra/Ra_{c}\right)}^{3/2}$ is known as *diffusion‐free scaling*. The physics of the diffusion‐free scaling, similar to the ultimate regime in RB convection (Grossmann & Lohse, 2011; Kraichnan, 1962; Shraiman & Siggia, 1990; Spiegel, 1971), is that the thermal and kinetic boundary layers, and thus the kinematic viscosity and thermal diffusivity, do not play an explicit role anymore for the heat flux scaling. This is known as bulk‐dominated convection.

So far, the diffusion‐free scaling has only been obtained in planar convection by considering an asymptotically reduced model in which Ekman pumping effects are not represented (Julien, Knobloch, et al., 2012) and numerical simulation with free‐stress boundaries and $Ek\leq 10^{-6}$ (Kunnen et al., 2016; Stellmach et al., 2014). For planar convection with no‐slip boundaries, King et al. (2012, 2013) theoretically predict α=3 for $Ra≲Ek^{-3/2}$. This finding follows from an analysis of the boundary layer stability and is supported by experimental and simulation data for $10^{-6}\leq Ek\leq \infty$. The difference between α=3 for no‐slip boundaries and α=3/2 for free‐stress boundaries is attributed to the active role of the Ekman pumping in the boundary layers near the plates (Julien et al., 2016; Plumley et al., 2016). However, the asymptotic diffusion‐free scaling exponent α=3/2 has not been reported for no‐slip boundaries in planar convection.

However, Gastine et al. (2016) find the diffusion‐free scaling for $Ek\leq 10^{-5}$ for $6Ra_{c}\leq Ra\leq 0.4Ek^{-8/5}$ in spherical RB convection with inner‐to‐outer radius ratio η=0.6 and no‐slip boundaries. The $Ek^{-8/5}$ scaling is proposed by Julien, Knobloch, et al. (2012); Julien, Rubio, et al. (2012). We note that previous theories of Gilman (1977) (giving the transitional Rayleigh number $Ra_{t}∼Ek^{-2}$ where $Ra_{t}$ represents for the upper bound of the diffusion‐free scaling region) and of King et al. (2009) (giving $Ra_{t}∼Ek^{-7/4}$) do not appropriately capture the upper bound of the diffusion‐free scaling region, which scales as $Ek^{-8/5}$.

The objective of this work is to elucidate the observation of diffusion‐free scaling in spherical RB convection at relatively weak rotation ($Ek∼10^{-5}$), while this scaling is not observed in planar convection. For strong rotation Ro≪1, the Taylor Proudman effect (Taylor, 1923) favors invariance along the rotation axis. In planar convection, see Figure 1a, the rotation axis is orthogonal to the plates, and the convective columns are homogeneously distributed in the horizontal direction and always stretch between the hot and cold plates. However, in spherical geometry, the rotation effect is latitude dependent; see Figure 1b, due to which three distinctly different flow regions are formed. Inside the inner sphere's tangent cylinder, the convective columns touch the inner and outer spherical boundaries. In the mid‐latitude region, the convective columns are stretched between the Northern and Southern hemispheres of the outer sphere. Near the equator, the convective columns adjust themselves to the curved boundary. This work will show that the diffusion‐free scaling originates from this mid‐latitude region. The article is organized as follows: In Section 2, we introduce the rotating spherical RB system with its control parameters. Section 3 is an overview of our simulation results compared and validated to literature, subsequent analysis is performed in Sections 4 and 5. Finally, we conclude our findings in Section 6.

## 2 Numerical Method, Control and Response Parameters

A sketch of the rotating spherical RB geometry is shown in Figure 1b. A fluid fills a spherical shell between the inner sphere of radius $r_{i}$ and outer sphere of radius $r_{o}$ with distance $d=r_{o}-r_{i}$ from the inner one. The whole system rotates about the vertical z axis at angular velocity Ω. The surface temperature of the inner and outer spheres is kept constant at $T_{i}$, and $T_{o}$, respectively, with $T_{i}>T_{o}$. No‐slip boundary conditions are imposed at both spheres. We solve the Navier‐Stokes equations in spherical coordinates within the Boussinesq approximation, which in dimensionless form read:

(1) $$\frac{\partial u}{\partial t}+u\cdot \nabla u=-\nabla p\;+\;\sqrt{\frac{Pr}{Ra}}\nabla^{2}u\;+\;gT{\vec{e}}_{r}-\frac{1}{Ro}{\vec{e}}_{z}\times u\;\;,\;\;\nabla \cdot u=0,$$

(2) $$\frac{\partial T}{\partial t}+u\cdot \nabla T=\frac{1}{\sqrt{RaPr}}\nabla^{2}T.$$

where $u\left(\vec{x},t\right)$, $p\left(\vec{x},t\right)$, $T\left(\vec{x},t\right)$, and g(r) denote the fluid velocity, pressure, temperature and radially dependent gravitational acceleration.

In this study, we focus on a radius ratio $\eta =r_{i}/r_{o}=0.6$ and the gravity profile $g\left(r\right)∼{\left(r_{o}/r\right)}^{2}$ valid for homogeneous mass distribution to allow comparisons with non‐rotating (Gastine et al., 2015) and rotating (Gastine et al., 2016) convection in spherical RB. This system configuration is considered representative for studying convection in gas giants (Long et al., 2020). Additionally, we perform simulations for η=0.35 and $g\left(r\right)∼{\left(r_{o}/r\right)}^{-1}$, which is considered an Earth‐like configuration used by Long et al. (2020) and Yadav et al. (2016). The equations are discretized by a staggered central second‐order finite‐difference scheme in spherical coordinates (Santelli et al., 2020). We use a uniform grid in the longitudinal and co‐latitudinal directions and ensure that the bulk and boundary layers are appropriately resolved (Stevens, Verzicco, & Lohse, 2010). The grid cells are clustered toward the inner and outer sphere to ensure the boundary layers are adequately resolved (Shishkina et al., 2010). Further details on the simulations are given in the Supporting Information S1.

The dynamics of rotating spherical RB convection are determined by the Rayleigh, Prandtl, and Ekman numbers:

(3) $$Ra=\frac{\beta g_{o}d^{3}\Delta T}{\kappa \nu },\;Pr=\frac{\nu }{\kappa },\;Ek=\frac{\nu }{\Omega d^{2}},$$

where β is the thermal expansion coefficient, $g_{o}$ is the gravity at the outer sphere, ν is the kinematic viscosity, and κ is the thermal diffusivity of the fluid. Ra is a measure of the thermal driving of the system, Ek characterizes the ratio of viscous to Coriolis forces, and Pr indicates the ratio of the viscous to thermal diffusivities. In this study we consider Pr=1. We use the Rossby number $Ro\equiv \sqrt{Ra/Pr}Ek/2$ to evaluate the relative importance of rotation and buoyancy (Gilman, 1977). We normalize the results using the length scale $d=r_{o}-r_{i}$, the temperature difference ΔT between inner and outer sphere, and the free‐fall velocity $U=\sqrt{\beta g_{o}\Delta Td}$.

The Nusselt number quantifies the non‐dimensional heat transport:

(4) $$Nu=\frac{\bar{\langle u_{r}{T\rangle }_{s}}-\kappa \partial_{r}\bar{\langle {T\rangle }_{s}}}{-\kappa \partial_{r}T_{c}},$$

where $T_{c}\left(r\right)=\eta /\;\left[{\left(1-\eta \right)}^{2}r\right]-\eta /\;\left(1-\eta \right)$ is the conductive temperature profile in spherical shells with constant temperature boundary conditions $T_{c}\left(r_{i}\right)=1$ and $T_{c}\left(r_{o}\right)=0$. The notations ${\langle \cdots \;\rangle }_{s}$ represents the average over a spherical surface with constant distance from the center, e.g., ${\langle T\rangle }_{s}=\frac{1}{4\pi }\int_{0}^{2\pi }\int_{0}^{\pi }T\left(\theta ,r,\varphi \right)\sin\varphi d\varphi d\theta$. Overbar $\bar{\cdots }$ corresponds to time averaging. In the following discussion, we will use Nu on the outer sphere as a function of the co‐latitude:

(5) $$Nu\left(\varphi \right)={\left.-\frac{1}{\eta }\frac{d\bar{{\langle T\rangle }_{\theta }}}{dr}\right|}_{r_{o}}$$

where ${\langle \ldots \rangle }_{\theta }$ represents the average over the azimuthal direction, e.g., ${\langle T\rangle }_{\theta }=\frac{1}{2\pi }\int_{0}^{2\pi }T\left(\theta ,r,\varphi \right)d\theta$.

## 3 Heat Transfer in Rotating Spherical RB Convection

**Figure 2:**
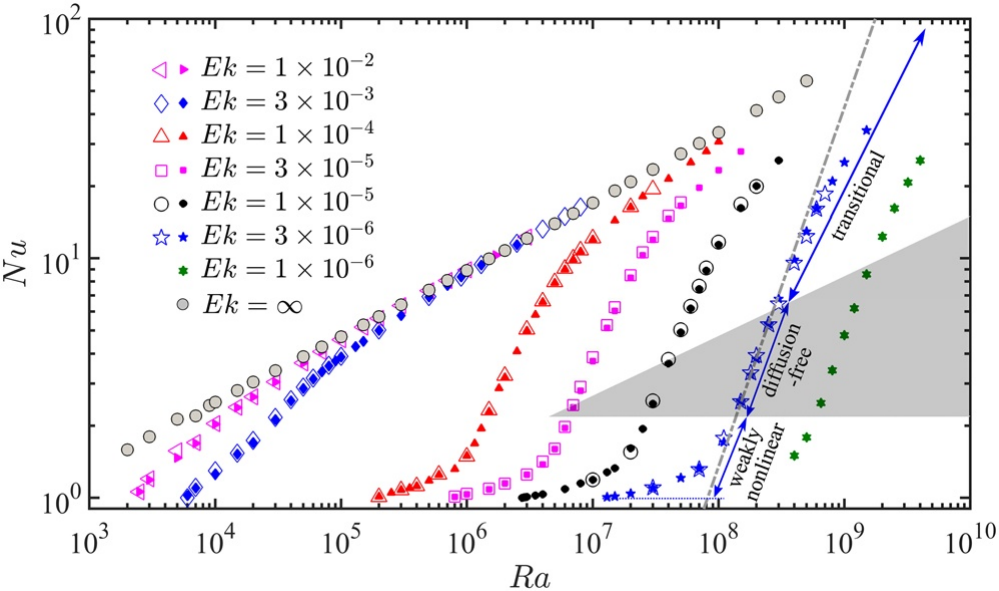
Nu as function of Ra for different Ek. Rotating cases: open symbols indicate the present results, filled‐in symbols are those from Gastine et al. (2016). Non‐rotating cases from Gastine et al. (2015) are indicated by Ek=∞. The shaded wedge‐shaped region indicates the diffusion‐free scaling regime ($6Ek^{4/3}\leq Ra\leq 0.4Ek^{-8/5}$), which corresponds to the quasi‐geostrophic regime identified by Gastine et al. (2016). The dot‐dashed gray line gives the diffusion‐free scaling $Nu=0.149R^{3/2}$ for $Ek=3\times 10^{-6}$. The error bars are smaller than the symbol sizes.

Figure 2 shows Nu as function of Ra for various Ek. The results from our simulations agree excellently with those from Gastine et al. (2016). For strong enough rotation (e.g., $Ek\leq 3\times 10^{-5}$), with increasing Ra three regimes can be identified (Gastine et al., 2016; Long et al., 2020). For low Ra, in the weakly nonlinear regime, rotational effects are dominant (Ro≪1) and $Nu∼R^{\alpha }$ with $R\equiv RaEk^{4/3}$ and α=1. In the quasi‐geostropic regime with diffusion‐free scaling α=3/2, the Taylor‐Proudman effect favors invariance along the rotation axis, thereby suppressing global heat transport relative to non‐rotating case (Julien, Knobloch, et al., 2012). This regime is observed for $6Ek^{4/3}\leq Ra\leq 0.4Ek^{-8/5}$ (Gastine et al., 2016). The lower bound is related to $Ra_{c}$, while the upper bound corresponds to the asymptotic prediction for bulk‐limited heat transfer in geostrophic turbulence by Julien, Knobloch, et al. (2012). In the transitional regime between strong and weak rotation (Ro∼1) the buoyancy force gradually becomes dominant over rotational effects with increasing Ra and the flow eventually approaches the non‐rotating case for Ro≫1.

## 4 Identification of Three Flow Regimes

**Figure 3:**
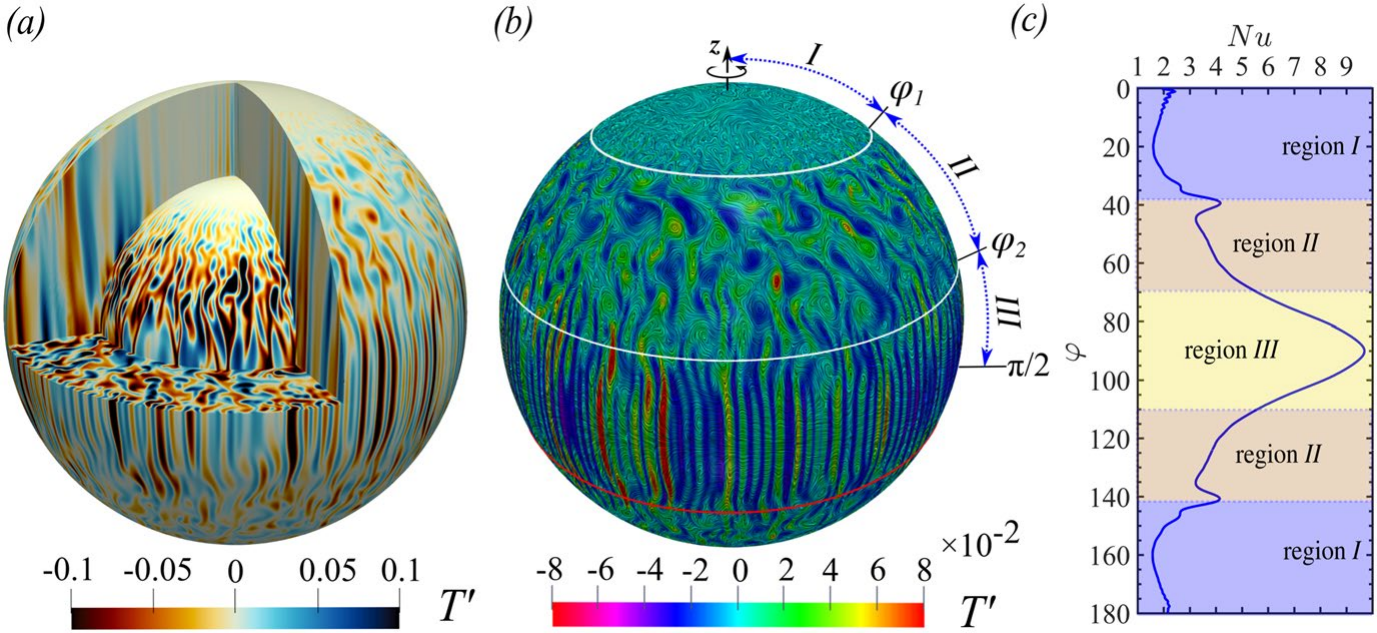
(a) Contour of the temperature fluctuation $T^{\prime }$ on two meridional cuts, equatorial section, and two spherical surfaces (corresponding to the spherical surfaces located at the inner ($r=r_{i}+\lambda_{T,i}$) and outer ($r=r_{o}-\lambda_{T,o}$) thermal boundary layers). (b) Contour of $T^{\prime }$ with streamlines illustrated by using line integral convolution on the outer radial surface (see Section 3 in the Supporting Information S1). The definition of the three regimes I,II,III is given in the text and Figure 4. (c) Nu as function of the co‐latitude φ on the outer sphere. In all cases (a–c), $Ek=1\times 10^{-5}$ and $Ra=5\times 10^{7}$, i.e., simulation No.76 in the Supporting Information S1.

Figure 3a visualizes the columnar structures by $T^{\prime }>0$ and $T^{\prime }<0$, here $T^{\prime }\left(\theta ,r,\varphi \right)=T\left(\theta ,r,\varphi \right)-\bar{{\langle T\rangle }_{s}}$, $\bar{{\langle T\rangle }_{s}}$ is defined in Section 2. The inner and outer thermal boundary layer thickness $\lambda_{T,i}$ and $\lambda_{T,o}$ is defined by the intersection of the linear fit to ${\langle T\rangle }_{s}$ near the boundaries and the profile at middepth (Gastine et al., 2016; Long et al., 2020). Figure 3b clearly shows that there are three distinct flow regions. Region I spans from the rotation axis to $\varphi_{1}$, where $\varphi_{1}$ can be determined by the intersection between the cylinder tangent to the inner sphere with the outer sphere. In this region, the columnar structures connect the boundary layers around the inner and outer spheres. Region II is found between $\varphi_{1}$ and $\varphi_{2}$ (see Figure 3b), $\varphi_{2}$ being the maximum zonal flow location (see below). In this mid‐latitude region, the structures are the strongest, and tall thin columns stretch from the Northern to the Southern parts of the cold outer sphere. Region III is the region around the equator, see Figure 3b. In this region, the structures aligned with the rotation axis are much shorter than in the mid‐latitude region II, while they conform themselves to the outer spherical boundary. Figure 3c shows that the heat transport strongly depends on the latitude (Yadav et al., 2016), which means that the heat transfer in the different flow regions identified above is different.

**Figure 4:**
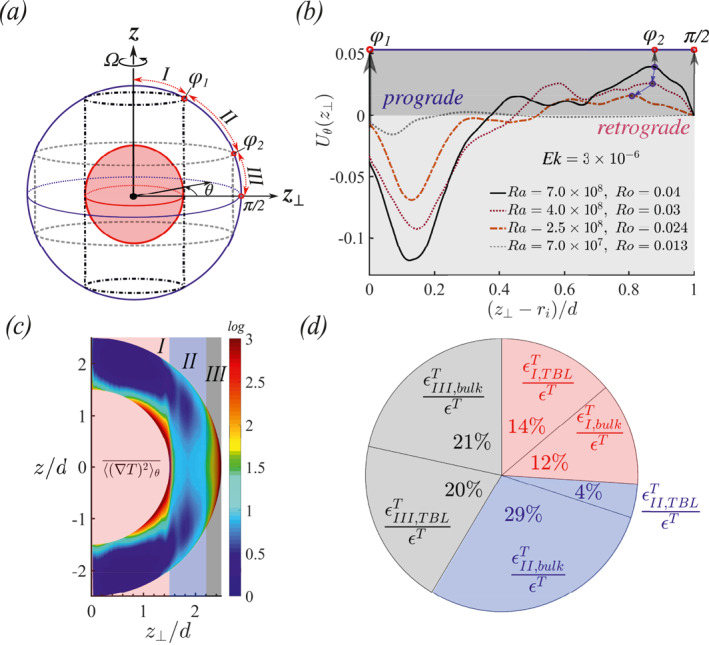
(a) Cylindrical polar coordinates $\left(z,z_{⊥},\theta \right)$, z is the rotation axis, $z_{⊥}$ is the cylindrical radius and θ is the azimuthal angle and of which the regimes I,II,III can be defined as shown. (b) Ensemble averaged azimuthal velocities $U_{\theta }$ (zonal flows) as a function of $z_{⊥}$ in Equation 6. $\left(z_{⊥}-r_{i}\right)\;/d=0$ and 1 correspond to the tangent cylinders of the inner and outer spheres, respectively. $\varphi_{2}$ is determined by the $z_{⊥}$ location close to the outer sphere ($\left(z_{⊥}-r_{i}\right)\;/d=1$) where the zonal flow is strongest. (c) Time and azimuthal averaged thermal dissipation $\bar{{\langle {\left(\nabla T\right)}^{2}\rangle }_{\theta }}$ in the meridional plane for case No.76 of $Ek=1\times 10^{-5}$ and $Ra=5\times 10^{7}$. (d) Pie chart for (c) showing the distribution of the thermal dissipation rate over the different regions in the boundary layer and bulk, see Equation 8.

Aurnou and Olson (2001) and Christensen (2002) found that the zonal flow is prograde in the equatorial region near the outer boundary and retrogrades near the tangent cylinder that encloses the central core. Therefore, the zonal flow is suitable to identify the boundary between region II and III. Figures 4a and 4b show how we use the local maximum prograde zonal velocity close to the equator to set $\varphi_{2}$. Figure 4a illustrates the cylindrical coordinate system $\left(z,z_{⊥},\theta \right)$ that is used to represent the zonal flow in Figure 4b. The zonal flow is the ensemble average of the azimuthal velocity in cylindrical coordinate:

(6) $$U_{\theta }\left(z_{⊥}\right)=\bar{{\langle u_{\theta }\left(z,z_{⊥},\theta \right)\rangle }_{\theta ,z}}$$

where $u_{\theta }\left(z,z_{⊥},\theta \right)$ is the longitudinal velocity $u_{\theta }\left(\theta ,r,\phi \right)$ in spherical coordinate projected to cylindrical coordinate, ${\langle \cdots \;\rangle }_{\theta ,z}$ indicates spatial average over a cylindrical surface (in the azimuthal and vertical direction), and $\bar{\cdots }$ indicates time‐averaging.

We analyze the thermal dissipation in the different flow regions to determine whether the different regions are dominated by the boundary layer or the bulk dynamics. For spherical shells with radius ratio η, the thermal dissipation rate:

(7) $$\epsilon^{T}\equiv \bar{\langle {\left(\nabla T\right)}^{2}\rangle }=\frac{3\eta }{1+\eta +\eta^{2}}Nu$$

by volume integral of T× (2). Figure 4c shows the time‐averaged thermal dissipation rate in the meridional plane. The figure shows that the thermal dissipation intensity is highest in the boundary layers along the inner sphere (region I) and close to the equator region along the outer sphere (region III). We determine the distribution of the thermal dissipation rate over the different regions as follows:

(8) $$\epsilon^{T}=\epsilon_{I,bulk}^{T}+\epsilon_{I,TBL}^{T}+\epsilon_{II,bulk}^{T}+\epsilon_{II,TBL}^{T}+\epsilon_{III,bulk}^{T}+\epsilon_{III,TBL,}^{T}$$

where bulk indicates the bulk regions and TBL indicates the thermal boundary layer regions, i.e., for the radial locations *r*; $r_{i}\leq r\leq r_{i}+\lambda_{T,i}$ along the inner sphere and $r_{o}-\lambda_{T,o}\leq r\leq r_{o}$ along the outer sphere. Figure 4d confirms that regions I and III are both strongly affected by the boundary layer dynamics. However, region II turns out to be bulk‐dominated. We note that the boundary between region II and III is not determined based on the thermal dissipation profiles as there is not a clear peak in the direction separating the regimes. Therefore, as discussed above, we use the maximum in the zonal flow profile to determine this transition.

In the following section, we will show that, in agreement with theoretical expectations discussed above, the scaling of the heat transfer in the region II follows the diffusion‐free scaling for rotation dominated strongly thermally driven flows.

## 5 Diffusion‐Free Scaling in Region II

**Figure 5:**
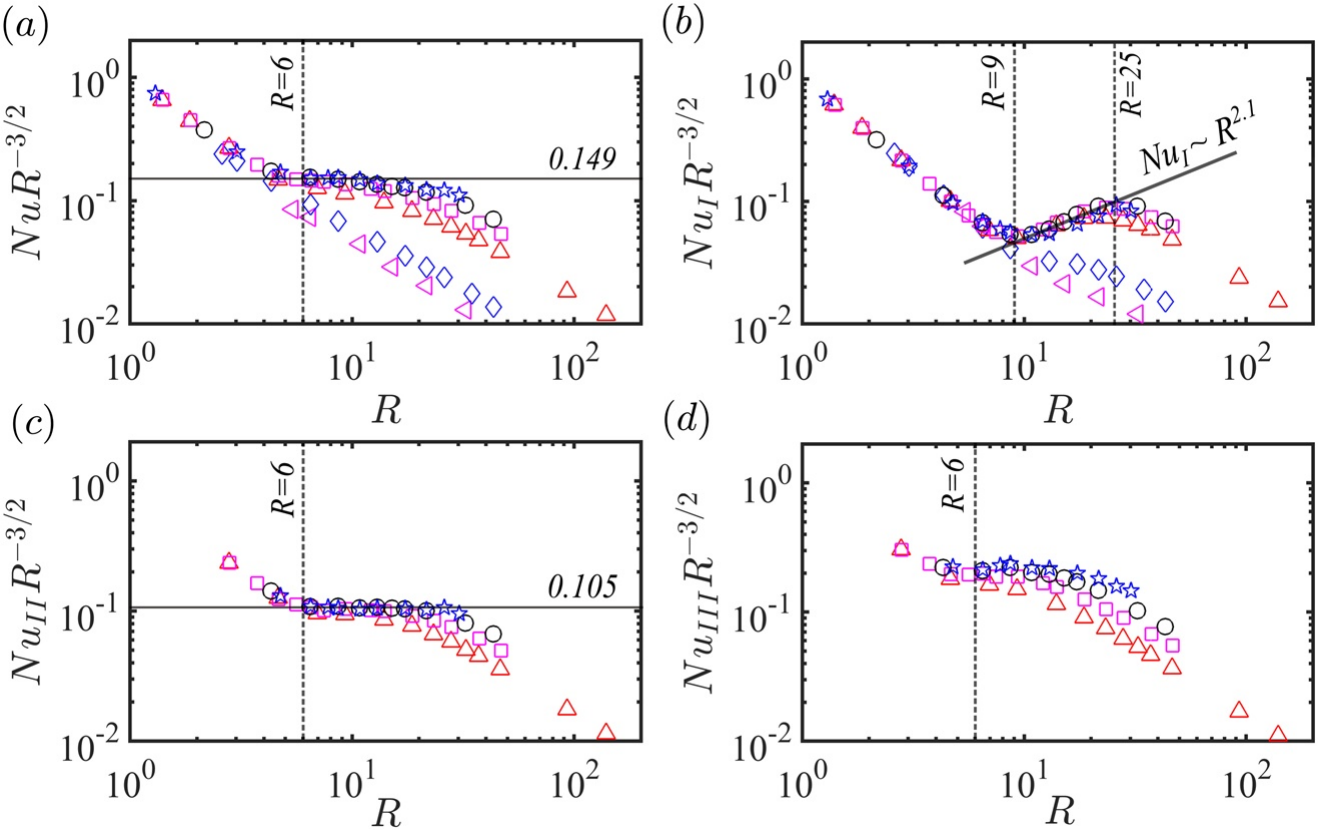
Nu on the outer sphere compensated by $R^{-3/2}$ and as a function of $R\equiv RaEk^{4/3}$. (a) Integration over the whole sphere; (b–d) Nu in regions (I−III), see Figure 3b. The symbols have the same meaning as in Figure 2.

Figure 5 shows Nu on the outer sphere compensated with the diffusion‐free scaling law. Panel 5(a) shows that for the global heat transfer and $Ek\leq 5\times 10^{-5}$ the diffusion‐free scaling is observed for R≥6. The crossover from the quasi‐geostrophic region to the transitional region is observed at $Ra_{t}=0.4Ek^{-8/5}$ (Gastine et al., 2016). Figures 5b–5d show the heat transfer scaling in the different flow regions identified above. Panel 5(b) evidences that, due to Ekman pumping (Stellmach et al., 2014; Stevens, Clercx, & Lohse, 2010; Stevens et al., 2013; Zhong et al., 2009), the heat transport scaling in region I is $Nu_{I}∼R^{2.1}$. This is steeper than the α=3/2 scaling for diffusion‐free convection, but shallower than the α=3 value observed in planar convection (King et al., 2013). Most importantly, panel 5(c) shows that the diffusion‐free scaling is much more pronounced in region II than in region I. Although the diffusion‐free scaling still starts at R=6, it continues for much higher R than the global heat transfer, see Figure 5a. Panel 5(d) shows that no diffusion‐free scaling regime is observed in region III.

**Figure 6:**
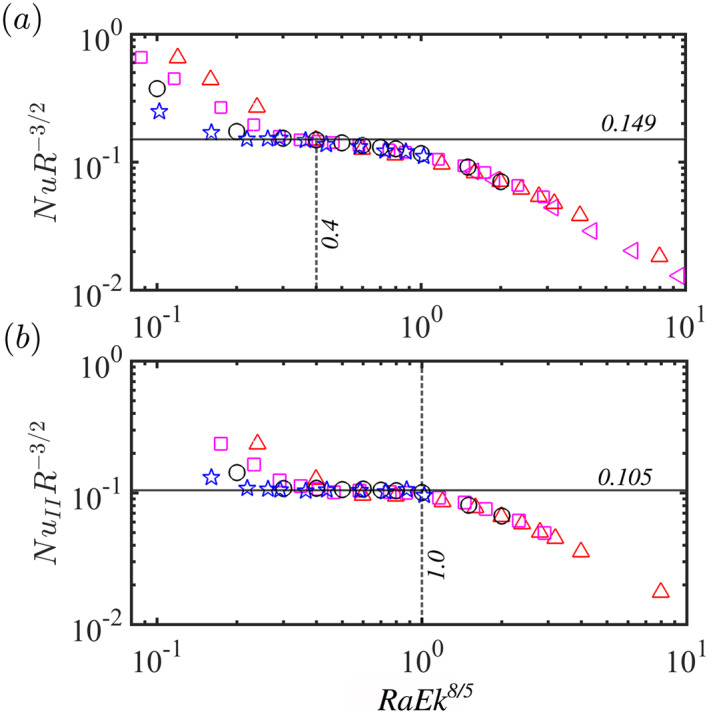
Nu compensated by $R^{-3/2}$ as a function of $RaEk^{8/5}$. (a) Integration over the whole sphere. The horizontal line is $NuR^{-3/2}=0.149$ and the vertical line is $RaEk^{8/5}=0.4$; (b) Region II. The horizontal line is $NuR^{-3/2}=0.105$ and the vertical line is $RaEk^{8/5}=1$. The symbols have the same meaning as in Figure 2.

The diffusion‐free scaling regime is observed from 6R up to $Ra_{t}$, where $Ra_{t}$ indicates the Ra number at which the regime for bulk‐limited heat transfer in geostrophic turbulence ends (Julien, Knobloch, et al., 2012; Julien, Rubio, et al., 2012). It was demonstrated (Gastine et al., 2016) that for the global heat transfer the diffusion‐free scaling regime is observed up to $Ra_{t}=0.4Ek^{-8/5}$, see also Figure 6a. For region II, Figure 6b shows that the diffusion‐free scaling is observed up to $Ra_{t}=Ek^{-8/5}$, which is considerably higher Ra than for the global heat transport.

In Section 4 of the Supporting Information S1, we show that the observation of the diffusion‐free scaling in the mid‐latitude region II does not depend on the specific η=0.6, $g\left(r\right)∼{\left(r_{o}/r\right)}^{2}$ considered here. The same conclusion is obtained by analyzing η=0.35, $g\left(r\right)∼{\left(r_{o}/r\right)}^{-1}$ and $Ek=1\times 10^{-5}$.

## 6 Conclusions

In conclusion, we have shown that rotating spherical RB convection has three distinctly different flow regions; see Figure 3b. In region I, convective columns are formed between the hot inner and cold outer spheres. The mid‐latitude region II is the region where the vertically aligned vortices are strongest, and the flow is bulk dominated. Region III is formed around the equator, and here the vortices are shorter and are affected by the outer spherical boundary.

The diffusion‐free scaling $Nu∼{\left(RaEk^{4/3}\right)}^{\alpha }$ with α=3/2 originates from the mid‐latitude flow region in which the flow dynamics are bulk dominated. In this region, thin and long convective columns are formed between the Northern and Southern parts of the cold outer sphere. This geostrophically dominated flow region can be formed due to the system geometry. Due to the curvature effects in spherical geometries, the latitude‐dependent Coriolis force results in inhomogeneous convective columns in the co‐latitudinal direction and more convective columns on the outer sphere than the inner sphere.

## Supporting information

Supporting Information S1

## Data Availability

The data used in this article are available for download at https://doi.org/10.5281/zenodo.5034407.
