# Supplementary material for: Diffusion‐Free Scaling in Rotating Spherical Rayleigh‐Bénard Convection
Source: Geophys Res Lett. 2021 Oct 21;48(20):e2021GL095017. doi: 10.1029/2021GL095017 (PMC9285093; doi:10.1029/2021GL095017)
Supplement: Supplementary file 1 — Supporting Information S1 [file GRL-48-0-s001.pdf]

Supporting Information for "Diffusion-free scaling in rotating spherical Rayleigh-Bénard convection"

Guiquan Wang[1], Luca Santelli[3], Detlef Lohse[1,4], Roberto Verzicco[1,2,3],

Richard J. A. M. Stevens[1]

[1]Physics of Fluids Group and Twente Max Planck Center, Department of Science and Technology, Mesa+ Institute, and J. M. Burgers Center for Fluid Dynamics, University of Twente, P.O. Box 217, 7500 AE Enschede, The Netherlands

[2]Dipartimento di Ingegneria Industriale, University of Rome' Tor Vergata', Via del Politecnico 1, 00133 Rome, Italy

[3]Gran Sasso Science Institute, Viale F. Crispi 7, 67100 L'Aquila, Italy

[4]Max Planck Institute for Dynamics and Self-Organization, Am Fassberg 17, 37077 Göttingen, Germany

Contents of this file

1. Text S1 to S4
2. Figures S1 to S5
3. Tables S1 to S7

Text S1. Simulation details

We use the conduction state $T(\theta, r, \varphi) = T_c(r)$ and $\mathbf{u} = 0$ as the initial conditions for the simulations. Simulations of $1-94$ are run at $Pr = 1$, $\eta = 0.6$ and $g \sim (r_o/r)^{n_g}$ with $n_g = 2$. Simulations of $95-111$ are run at $Pr = 1$, $\eta = 0.35$ and $n_g = -1$. The columns from left to right indicate: case number, Rayleigh number Ra, Rossby number Ro, the number of grid points in the longitudinal, radial, and co-latitudinal direction $N_\theta \times N_r \times N_\varphi$. A rotational symmetry order n_s is applied to reduce computational costs, indicating the longitude of the computational domain as $2\pi/n_s$. The average heat transfer Nu across the inner and the outer sphere, and the regional heat transfer across the outer sphere $Nu_{I,II,III}$ (see figure 3(b) of the paper). Note that region II and III are only defined when a maximum prograde velocity is identified.

Table S1: $Ek = 1 \times 10^{-2}, \eta = 0.6, n_g = 2$

	Ra	Ro	$N_\theta \times N_r \times N_\varphi$	n_s	Nu	Nu_{I}	Nu_{II}	Nu_{III}	φ_2
1	2.5×10^3	0.25	$257 \times 71 \times 161$	1	1.058	1.029	–	–	–
2	3×10^3	0.27	$257 \times 71 \times 161$	1	1.200	1.026	–	–	–
3	5×10^3	0.35	$257 \times 71 \times 161$	1	1.566	1.050	–	–	–
4	7×10^3	0.42	$257 \times 71 \times 161$	1	1.703	1.242	–	–	–
5	1×10^4	0.50	$257 \times 71 \times 161$	1	2.036	1.662	–	–	–
6	1.5×10^4	0.61	$257 \times 71 \times 161$	1	2.390	2.205	–	–	–
7	2×10^4	0.71	$257 \times 71 \times 161$	1	2.637	2.521	–	–	–
8	3×10^4	0.87	$257 \times 71 \times 161$	1	3.050	2.952	–	–	–
9	5×10^4	1.12	$257 \times 71 \times 161$	1	3.661	3.443	–	–	–
10	7×10^4	1.32	$257 \times 71 \times 161$	1	4.080	3.710	–	–	–
11	1×10^5	1.58	$401 \times 81 \times 257$	1	4.566	4.055	–	–	–
12	1.5×10^5	1.94	$401 \times 81 \times 257$	1	5.155	4.504	–	–	–
13	2×10^5	2.24	$401 \times 81 \times 257$	1	5.608	4.749	–	–	–
14	3×10^5	2.74	$401 \times 81 \times 257$	1	6.335	5.378	–	–	–
15	5×10^5	3.54	$401 \times 81 \times 257$	1	7.322	6.098	–	–	–
16	7×10^5	4.18	$401 \times 81 \times 257$	1	8.074	6.745	–	–	–
17	1×10^6	5.00	$401 \times 81 \times 257$	1	8.952	7.490	–	–	–
18	3×10^6	8.66	$601 \times 101 \times 401$	1	12.251	10.685	–	–	–

Table S2: $Ek = 3 \times 10^{-3}, \eta = 0.6, n_g = 2$

	Ra	Ro	$N_\theta \times N_r \times N_\varphi$	n_s	Nu	Nu_{I}	Nu_{II}	Nu_{III}	φ_2
19	6×10^3	0.12	$257 \times 71 \times 161$	1	1.000	1.030	–	–	–
20	7×10^3	0.13	$257 \times 71 \times 161$	1	1.105	1.020	–	–	–
21	1×10^4	0.15	$257 \times 71 \times 161$	1	1.297	1.007	–	–	–
22	1.5×10^4	0.18	$257 \times 71 \times 161$	1	1.534	1.004	–	–	–
23	2×10^4	0.21	$257 \times 71 \times 161$	1	1.734	1.049	–	–	–
24	3×10^4	0.26	$257 \times 71 \times 161$	1	2.164	1.517	–	–	–
25	4×10^4	0.30	$257 \times 71 \times 161$	1	2.571	2.211	–	–	–
26	5×10^4	0.34	$257 \times 71 \times 161$	1	2.895	2.781	–	–	–
27	6×10^4	0.37	$257 \times 71 \times 161$	1	3.157	3.223	–	–	–
28	8×10^4	0.42	$257 \times 71 \times 161$	1	3.577	3.884	–	–	–
29	1×10^5	0.47	$321 \times 91 \times 201$	1	3.895	4.334	–	–	–
30	2×10^5	0.67	$321 \times 91 \times 201$	1	5.021	5.634	–	–	–
31	5×10^5	1.06	$321 \times 91 \times 201$	1	6.916	7.127	–	–	–
32	9×10^5	1.42	$451 \times 101 \times 301$	1	8.347	8.226	–	–	–
33	1.3×10^6	1.71	$451 \times 101 \times 301$	1	9.421	8.973	–	–	–
34	2.5×10^6	2.37	$451 \times 101 \times 301$	1	11.549	10.267	–	–	–
35	4×10^6	3.00	$301 \times 121 \times 401$	2	13.238	11.788	–	–	–
36	6×10^6	3.67	$301 \times 121 \times 401$	2	14.960	13.580	–	–	–
37	8×10^6	4.24	$301 \times 121 \times 401$	2	16.369	15.571	–	–	–

Table S3: $Ek = 1 \times 10^{-4}, \eta = 0.6, n_g = 2$

	Ra	Ro	$N_\theta \times N_r \times N_\varphi$	n_s	Nu	Nu_{I}	Nu_{II}	Nu_{III}	φ_2
38	2×10^5	0.02	$145 \times 73 \times 217$	4	1.008	1.021	–	–	–
39	3×10^5	0.03	$145 \times 73 \times 217$	4	1.077	1.018	–	–	–
40	4×10^5	0.03	$145 \times 73 \times 217$	4	1.119	1.012	–	–	–
41	6×10^5	0.04	$145 \times 73 \times 217$	4	1.250	1.008	1.096	1.426	0.92
42	1×10^6	0.05	$257 \times 81 \times 401$	4	1.488	1.009	1.269	1.807	1.00
43	1.5×10^6	0.06	$257 \times 81 \times 401$	4	2.317	1.070	1.770	2.999	0.97
44	2×10^6	0.07	$257 \times 81 \times 401$	4	3.233	1.420	2.680	4.261	1.01
45	3×10^6	0.09	$257 \times 81 \times 401$	4	5.038	3.318	4.472	5.992	0.98
46	4×10^6	0.10	$257 \times 81 \times 401$	4	6.613	5.844	6.184	7.278	1.00
47	5×10^6	0.11	$257 \times 81 \times 401$	4	7.949	8.242	7.414	8.367	1.05
48	6×10^6	0.12	$257 \times 81 \times 401$	4	9.060	10.233	8.572	9.147	1.04
49	7×10^6	0.13	$257 \times 81 \times 401$	4	9.996	11.913	9.328	9.941	1.08
50	8×10^6	0.14	$257 \times 81 \times 401$	4	10.801	13.309	10.246	10.532	1.07
51	1×10^7	0.16	$257 \times 81 \times 401$	4	12.110	15.364	11.286	11.608	1.11
52	2×10^7	0.22	$257 \times 101 \times 401$	4	16.381	21.292	15.746	15.167	1.10
53	3×10^7	0.27	$257 \times 101 \times 401$	4	19.410	25.163	18.848	18.105	1.08

Table S4: $Ek = 3 \times 10^{-5}, \eta = 0.6, n_g = 2$

	Ra	Ro	$N_\theta \times N_r \times N_\varphi$	n_s	Nu	Nu_{I}	Nu_{II}	Nu_{III}	φ_2
54	8×10^5	0.013	$145 \times 73 \times 217$	4	1.011	1.022	–	–	–
55	1×10^6	0.015	$145 \times 73 \times 217$	4	1.039	1.020	–	–	–
56	1.5×10^6	0.018	$145 \times 73 \times 217$	4	1.090	1.016	–	–	–
57	2×10^6	0.021	$145 \times 73 \times 217$	4	1.148	1.011	–	–	–
58	3×10^6	0.026	$145 \times 73 \times 217$	4	1.251	1.008	1.104	1.425	0.921
59	4×10^6	0.030	$145 \times 73 \times 217$	4	1.418	1.002	1.185	1.710	0.968
60	5×10^6	0.034	$289 \times 73 \times 401$	4	1.604	1.006	1.251	1.985	0.961
61	6×10^6	0.037	$289 \times 73 \times 401$	4	1.978	1.014	1.490	2.591	0.993
62	7×10^6	0.040	$289 \times 73 \times 401$	4	2.435	1.061	1.785	3.316	1.006
63	8×10^6	0.042	$289 \times 81 \times 401$	4	2.905	1.148	2.062	3.838	0.940
64	1×10^7	0.047	$289 \times 81 \times 401$	4	3.865	1.479	2.959	5.351	1.026
65	1.3×10^7	0.054	$289 \times 81 \times 401$	4	5.268	2.495	4.302	7.067	1.050
66	1.5×10^7	0.058	$289 \times 81 \times 401$	4	6.251	3.514	5.245	8.221	1.078
67	2×10^7	0.067	$289 \times 103 \times 433$	4	8.491	6.657	7.414	10.115	1.066
68	2.5×10^7	0.075	$289 \times 103 \times 433$	4	10.599	9.998	9.540	11.863	1.081
69	3×10^7	0.082	$289 \times 103 \times 433$	4	12.294	12.861	11.164	13.391	1.125
70	4×10^7	0.095	$321 \times 103 \times 481$	4	15.054	16.930	14.064	15.427	1.120
71	5×10^7	0.106	$321 \times 103 \times 481$	4	17.148	19.940	15.847	17.549	1.158

Table S5: $Ek = 1 \times 10^{-5}, \eta = 0.6, n_g = 2$

	Ra	Ro	$N_\theta \times N_r \times N_\varphi$	n_s	Nu	Nu_{I}	Nu_{II}	Nu_{III}	φ_2
72	1×10^7	0.016	$201 \times 91 \times 401$	8	1.190	1.007	–	–	–
73	2×10^7	0.022	$201 \times 91 \times 401$	8	1.554	1.001	1.280	1.987	1.059
74	3×10^7	0.027	$201 \times 91 \times 401$	8	2.537	1.097	1.780	3.449	0.988
75	4×10^7	0.032	$201 \times 91 \times 401$	8	3.784	1.363	2.750	5.607	1.076
76	5×10^7	0.035	$201 \times 91 \times 401$	8	5.097	1.897	3.754	7.169	1.055
77	6×10^7	0.039	$241 \times 96 \times 481$	8	6.296	2.757	4.970	9.120	1.132
78	7×10^7	0.042	$241 \times 96 \times 481$	8	7.644	3.995	6.166	10.746	1.129
79	8×10^7	0.045	$241 \times 96 \times 481$	8	9.114	5.585	7.472	12.311	1.119
80	1×10^8	0.050	$289 \times 115 \times 577$	8	11.697	9.153	10.089	14.710	1.138
81	1.5×10^8	0.061	$289 \times 115 \times 577$	8	16.815	16.786	14.865	18.800	1.138
82	2×10^8	0.071	$325 \times 130 \times 649$	8	19.973	19.499	18.912	21.855	1.114

Table S6: $Ek = 3 \times 10^{-6}, \eta = 0.6, n_g = 2$

	Ra	Ro	$N_\theta \times N_r \times N_\varphi$	n_s	Nu	Nu_{I}	Nu_{II}	Nu_{III}	φ_2
83	3×10^7	0.008	$241 \times 97 \times 481$	8	1.101	1.009	–	–	–
84	7×10^7	0.013	$241 \times 97 \times 481$	8	1.312	1.022	–	–	–
85	1.1×10^8	0.016	$241 \times 97 \times 481$	8	1.770	1.006	1.364	2.332	1.021
86	1.5×10^8	0.018	$289 \times 115 \times 577$	8	2.518	1.134	1.798	3.527	1.062
87	1.8×10^8	0.020	$325 \times 131 \times 649$	8	3.322	1.297	2.327	4.970	1.097
88	2×10^8	0.021	$325 \times 131 \times 649$	8	3.928	1.419	2.689	6.035	1.102
89	2.5×10^8	0.024	$385 \times 155 \times 769$	8	5.273	1.910	3.694	7.840	1.076
90	3×10^8	0.026	$385 \times 155 \times 769$	8	6.449	2.551	4.930	10.193	1.174
91	4×10^8	0.030	$433 \times 173 \times 867$	8	9.537	4.694	7.524	14.390	1.181
92	5×10^8	0.034	$433 \times 173 \times 867$	8	12.353	7.674	10.293	18.251	1.237
93	6×10^8	0.037	$433 \times 173 \times 867$	8	16.036	12.334	13.775	20.935	1.229
94	7×10^8	0.040	$433 \times 173 \times 867$	8	18.554	13.882	15.994	24.444	1.246

Table S7: $Ek = 1 \times 10^{-5}, \eta = 0.35, n_g = -1$

	Ra	Ro	$N_\theta \times N_r \times N_\varphi$	n_s	Nu	Nu_{I}	Nu_{II}	Nu_{III}	φ_2
95	2×10^7	0.022	$301 \times 73 \times 201$	2	1.101	1.010	–	–	–
96	3×10^7	0.027	$301 \times 73 \times 201$	2	1.224	1.010	–	–	–
97	4×10^7	0.032	$301 \times 73 \times 201$	2	1.360	1.011	–	–	–
98	5×10^7	0.035	$301 \times 73 \times 201$	2	1.503	1.010	–	–	–
99	6×10^7	0.039	$301 \times 73 \times 201$	2	1.639	1.012	–	–	–
100	7×10^7	0.042	$451 \times 93 \times 301$	2	1.793	1.011	–	–	–
101	8×10^7	0.045	$451 \times 93 \times 301$	2	1.909	1.001	–	–	–
102	9×10^7	0.047	$451 \times 93 \times 301$	2	2.168	1.014	–	–	–
103	1×10^8	0.050	$451 \times 93 \times 301$	2	2.436	1.022	1.769	4.223	1.256
104	1.4×10^8	0.059	$451 \times 93 \times 301$	2	3.458	1.149	2.186	6.567	1.250
105	1.8×10^8	0.067	$451 \times 93 \times 301$	2	4.844	1.874	3.096	9.110	1.247
106	2×10^8	0.071	$451 \times 93 \times 301$	2	5.587	2.666	3.591	9.924	1.229
107	2.5×10^8	0.079	$541 \times 103 \times 361$	2	7.234	5.701	4.881	11.187	1.193
108	3×10^8	0.087	$541 \times 103 \times 361$	2	9.043	10.791	6.546	12.822	1.203
109	4×10^8	0.10	$541 \times 103 \times 361$	2	12.029	18.876	9.546	14.401	1.216
110	6×10^8	0.122	$541 \times 103 \times 361$	2	17.171	27.367	15.087	18.196	1.244
111	8×10^8	0.141	$601 \times 103 \times 361$	2	21.525	31.511	20.098	23.118	1.246

Text S2. Grid independence and longitudinal domain size

A grid resolution study for case No.76 with $n_s = 8$ is presented in figure S1. In rapidly rotating spherical Rayleigh-Bénard (RB) flow, the convective columns are with length

scale $Ek^{1/3}d$ (Gastine et al., 2016), distributed along the longitudinal direction. This explains the large number of grids in the longitudinal direction, which is proportional to $Ek^{1/3}$(Gastine et al., 2016). In our numerical scheme the Nu number is sensitive to the grid resolution. The effect of the grid resolution in the longitude, radial, and co-latitude direction is shown in figure S1(a-c). We also verified to convergence by increasing the resolution in all directions simultaneously, i.e. from $201 \times 91 \times 401$ ($Nu = 5.097$) to $251 \times 111 \times 451$ ($Nu = 5.103$). Furthermore, we verified that the temperature (T_{rms}) and velocity ($u_{r,rms}$) root-mean-square profiles, which are more sensitive to the grid resolution, are fully converged. Overall, the tests confirm that $N_\theta \times N_r \times N_\varphi = 201 \times 91 \times 401$ is sufficient resolution for case No.76. The resolution of the other cases is adjusted accordingly.

Al-Shamali, Heimpel, and Aurnou (2004) found that the critical azimuthal wavenumber is nearly independent of assumed longitudinal symmetry up to $n_s = 8$. We have verified that the heat transfer does not depend on the longitudinal symmetry up to $n_s = 8$. Moreover, figure S2 shows that the first order and second order temperature statistics are independent to n_s. Therefore, we use n_s up to eight for high rotation rates to reduce computational cost.

For case No.76 used for Figure 3(c) in the paper, we observe the onset of vertically aligned vortices in the region I, which are "cellular" convective structures (we refer to the review paper Aurnou et al. (2015) about the flow morphology). These cellulars result in regular oscillations of $Nu(\varphi)$ close to the poles. To exclude that the oscillations are caused by numerical instabilities we perform two additional simulations, one with a finer mesh in the co-latitudinal direction, and one with a smaller CFL number. Figure S3 shows that

the Nu profiles obtained from these additional cases agrees excellently with standard case No.76, which confirms the accuracy of the results.

Text S3. Line Integral Convolution for spherical surfaces

Line integral convolution (LIC) is a technique introduced by Cabral and Leedom (1993) for generating texture from vector data as shown in figure 3(b) in the paper. This technique was initially dedicated to a 2D vector on a flat surface, as shown in figure S4. The streaking patterns follow vector field tangents. Afterwards it has been extended to volume and arbitrary surfaces. For Figure 3(b) in the paper, we use a 3D vector field projection onto a spherical surface based on triangle tiling. An excellent technical and mathematical description of this approach can, for example, be found in section 3.4 of Hege and Polthier (2013).

Text S4. Diffusive free region for $\eta = 0.35$ and $n_g = -1$

The temperature fluctuation T' in the vicinity of the outer radial surface in figure S5(a) clearly shows three distinct flow regions from the pole to the equator. The Nu and Nu_{II} are shown in figure S5(b). Again we find the diffusion-free scaling is much more pronounced in region II, similar to what we report for $\eta = 0.6$ and $n_g = 2$. This confirms that the main findings are independent of the selected η and gravity profile.

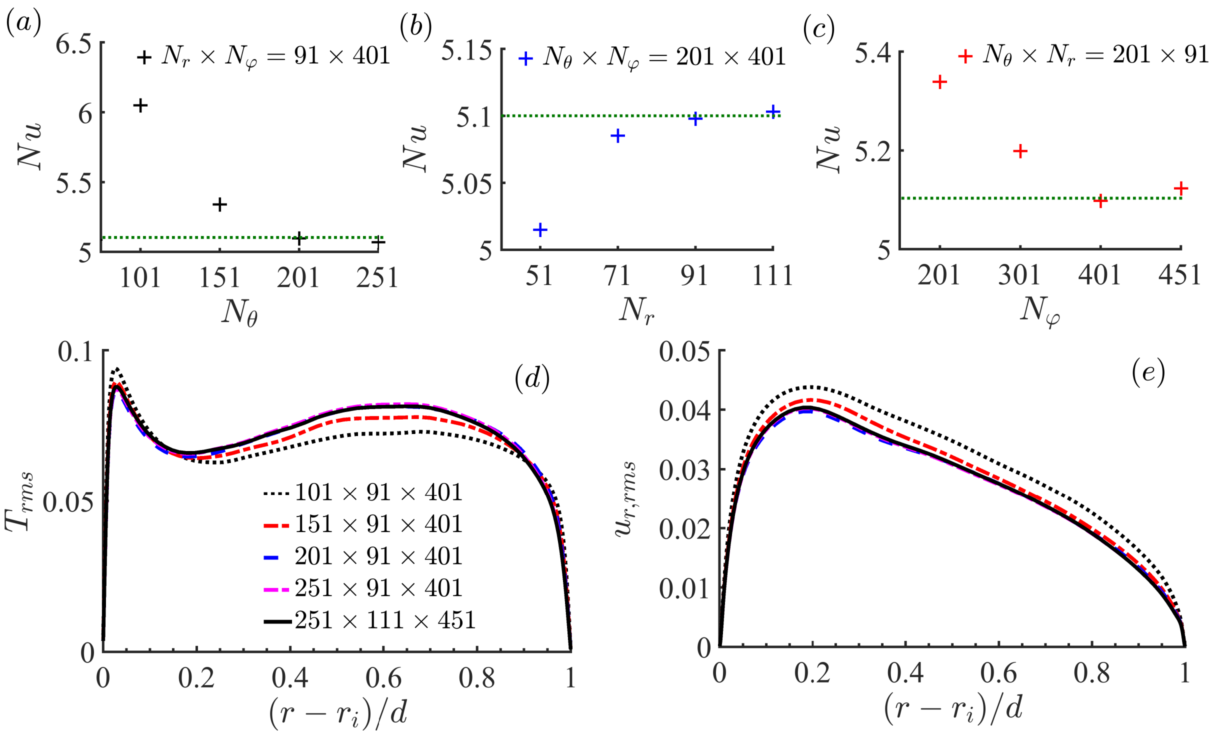

Figure S1. Grid convergence test for case No.76. Nu as function of (a) the longitudinal number of grid point N_θ; (b) the radial number of grid points N_r; (c) and the co-latitudinal number of grid points N_φ. The dotted line in (a-c) indicates $Nu = 5.103$ obtained using $N_\theta \times N_r \times N_\varphi = 251 \times 111 \times 451$. (d,e) Corresponding T_{rms} and $u_{r,rms}$ profiles as a function of radial direction, which show that higher order statistics are also converged.

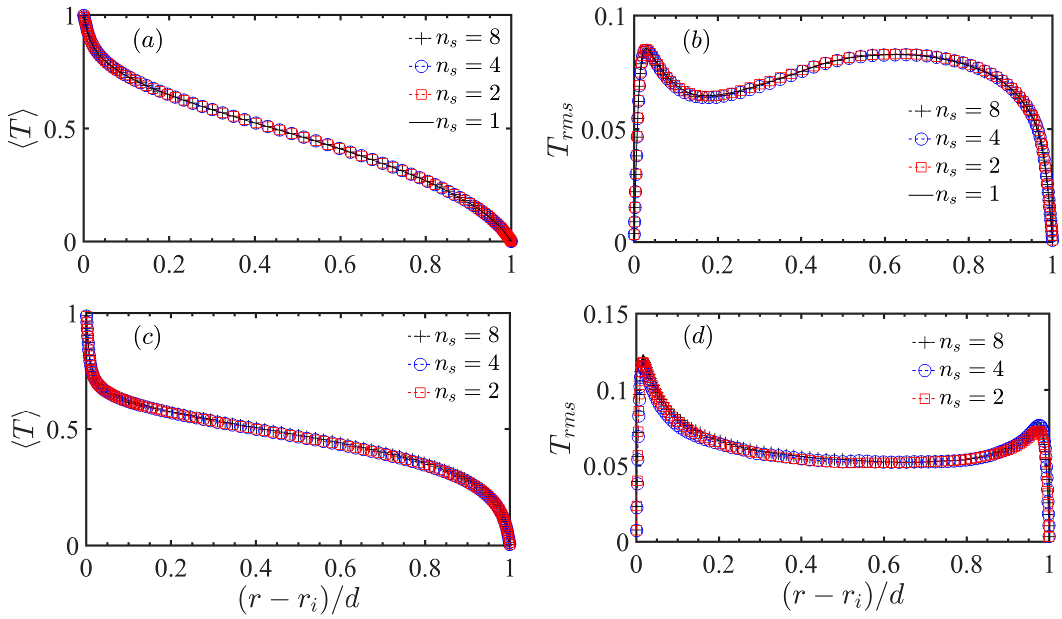

Figure S2. Number of rotational symmetry order n_s tests for $\langle T \rangle$ (a,c) and $\langle T_{rms} \rangle$ (b,d) as a function of radial direction for case No.76 (a,b) and case No.80 (c,d).

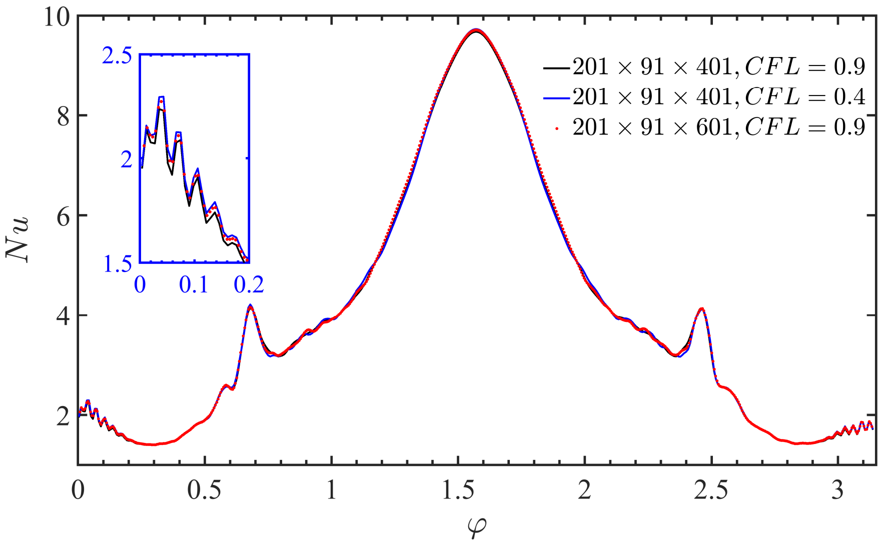

Figure S3. Resolution and CFL number tests for case No.76 of $Ek = 1 \times 10^{-5}$, $Ra = 5 \times 10^{7}$. Time and azimuthal averaged Nu as function of the co-latitude φ on the outer sphere.

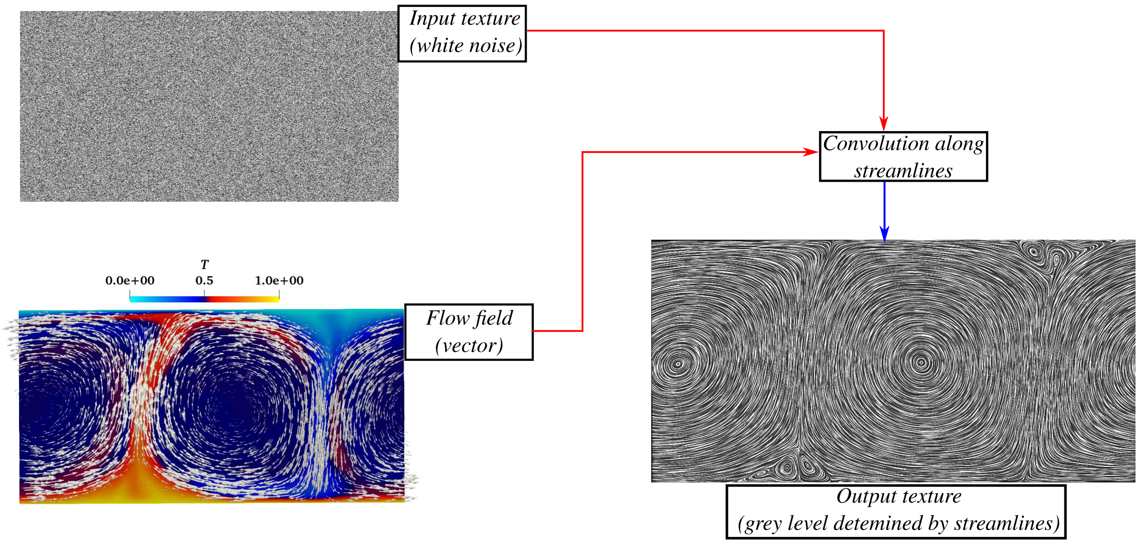

Figure S4. Schematic process of line integral convolution (LIC) computation for a 2D non-rotating RB flow: {vector field $\rightarrow$ streamline} + {input texture (white noise)} $\rightarrow$ {output texture (pixel grey level determined by the streamline)}.

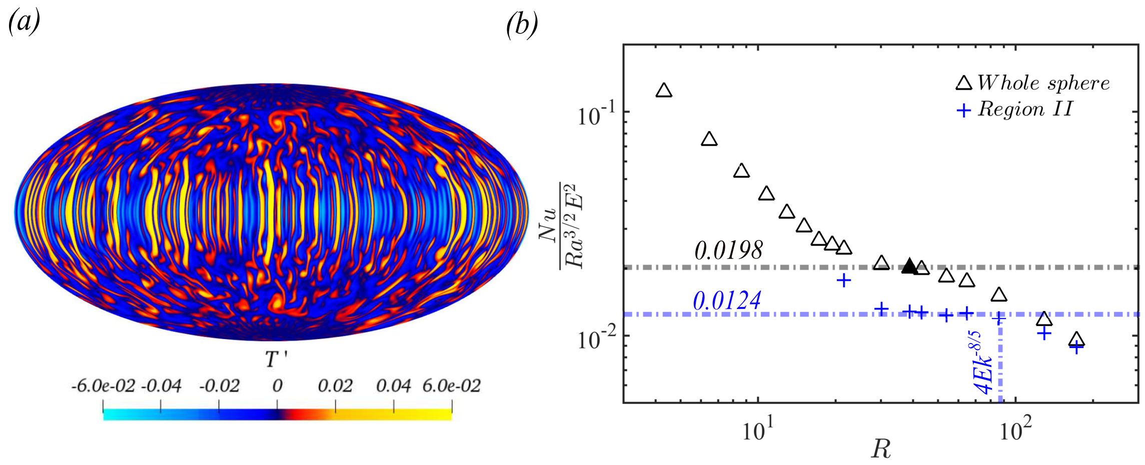

Figure S5. (a) Temperature fluctuation at the outer thermal boundary layer displayed in a Hammer projection for case No.105, corresponding to the filled-in symbol in (b). (b) Nu integration over the whole sphere and in region II on the outer sphere compensated by $R^{-3/2}$ and as a function of $R \equiv RaEk^{4/3}$.

References

Al-Shamali, F. M., Heimpel, M. H., & Aurnou, J. M. (2004). Varying the spherical shell geometry in rotating thermal convection. *Geophys. Astrophys. Fluid Dyn.*, *98*(2), 153-169.

Aurnou, J. M., Calkins, M. A., Cheng, J. S., Julien, K., King, E. M., Nieves, D., ... Stellmach, S. (2015). Rotating convective turbulence in Earth and planetary cores. *Phys. Earth Planet. Inter.*, *246*, 52–71.

Cabral, B., & Leedom, L. C. (1993). Imaging vector fields using line integral convolution. In *Proceedings of the 20th annual conference on computer graphics and interactive techniques* (pp. 263–270).

Gastine, T., Wicht, J., & Aurnou, J. M. (2016). Scaling regimes in spherical shell rotating convection. *J. Fluid Mech.*, *808*, 690–732.

Hege, H. C., & Polthier, K. (2013). *Visualization and mathematics iii.* Springer Science & Business Media.
